# The Characterization and Study of Antibacterial, Free Radical Scavenging, and Anticancer Potential of *Livistona chinensis*-Mediated Silver Nanoparticles

**DOI:** 10.3390/molecules28237773

**Published:** 2023-11-25

**Authors:** Aroona Saleem, Sikander Ali, Muhammad Nauman Aftab, Ashwag Shami, Fatimah A. Al-Saeed, Bilal Mustafa, Bilal Ahamad Paray

**Affiliations:** 1 Institute of Industrial Biotechnology (IIB), Government College University Lahore, Lahore 54000, Pakistan; aroonasaleem51@gmail.com (A.S.); dr.naumanaftab@gcu.edu.pk (M.N.A.); 2 Department of Biology, College of Science, Princess Nourah bint Abdulrahman University, Riyadh 11671, Saudi Arabia; 3 Department of Biology, College of Science, King Khalid University, Abha 61413, Saudi Arabia; 4 Wildlife Conservation Research Unit (WildCRU), Department of Biology, University of Oxford, Oxford OX13 5QL, UK; 5 Department of Zoology, College of Science, King Saud University, Riyadh 11451, Saudi Arabia

**Keywords:** *Livistona chinensis*, AgNPs, green synthesis, DPPH, reactive oxygen species, thyroid cancer cells

## Abstract

In the present research, *Livistona chinensis* leaf extracts were utilized as reductants to bio-fabricate silver nanoparticles (LC-AgNPs) and this was followed by the evaluation of their antioxidant, antibacterial, and anticancer potential. Multiple parameters were optimized for the formation and fidelity of LC-AgNPs. The color shift of the reaction mixture from yellow to dark brown confirmed the LC-AgNPs formation. UV/VIS spectroscopy exhibited a surface plasmon resonance (SPR) band at 436 nm. The Fourier transform infrared (FTIR) spectroscopy spectrum depicted phytochemicals in the plant extract acting as bio-reducers for LC-AgNPs synthesis. The XRD pattern confirmed the presence of LC-AgNPs by showing peaks corresponding to 2θ angle at 8.24° (111), 38.16° (200), 44.20° (220), and 64.72° (311). Zetasizer analysis exhibited size distribution by intensity of LC-AgNPs with a mean value of 255.7 d. nm. Moreover, the zeta potential indicated that the AgNPs synthesized were stable. The irregular shape of LC-AgNPs with a mean average of 38.46 ± 0.26 nm was found by scanning electron microscopy. Furthermore, the antioxidant potential of LC-AgNPs was examined using a DPPH assay and was calculated to be higher in LC-AgNPs than in leaf extracts. The calculated IC_50_ values of the LC-AgNPs and plant extract are 85.01 ± 0.17 and 209.44 ± 0.24, respectively. The antibacterial activity of LC-AgNPs was investigated against *Escherichia coli*, *Pseudomonas aeruginosa*, and *Bacillus subtilis* as well as *Staphylococcus aureus,* and maximum potential was observed after 24 h against *P. aeruginosa*. Moreover, LC-AgNPs exhibited maximum anticancer potential against TPC1 cell lines compared to the plant extract. The findings suggested that LC-AgNPs could be used as antioxidant, antibacterial, and anticancer agents for the cure of free-radical-oriented bacterial and oncogenic diseases.

## 1. Introduction

Nanotechnology has gained immense attention in the past era due to its applications in a wide range of fields [1,2]. The miniature size of nanoparticles (1–100 nm) has unlocked some excellent idiosyncratic properties, such as increased surface area, high strength, and toughness [3]. Silver nanoparticles (AgNPs) have acquired an exceptional space in the family of nanoparticles because of their broad spectrum of applications in electronics, catalysis, agriculture, textiles, food, waste management, and biomedicine [4,5,6]. They have been known for their extraordinary properties against bacteria, reactive oxygen species (ROS), and cancer cells. Silver nanoparticles have plasmon resonance peaks in the visible and near-infrared regions, which are highly desirable for many biological and medical applications; therefore, they have been preferred over the other nanoparticles for this research work [7]. They have been produced from different routes, including physical, chemical, and biological routes. However, plants have come forward as the most favorable candidates for their production, especially for AgNPs, for their enhanced scale-up capability, non-toxicity, cost-effectiveness, and simple synthesis method [8,9]. The plant extracts possess natural biochemical compounds with antibacterial, antioxidant, and anticancer capabilities [10,11,12]. For instance, in the past, AgNPs obtained from the leaf extract of *Morus alba* showed significant antineoplastic activity against MCF-7 cells and antibacterial properties against *Staphylococcus aureus*, *Acinetobacter baumannii*, *Escherichia coli*, *Pseudomonas aeruginosa*, and *Bacillus subtilis* [13]. Similarly, many biochemical compounds have been reported in the plants that possess antibacterial [14,15,16] as well as antioxidant [17,18] potential. This emphasizes the need to explore the plants for the production of AgNPs, as they possess natural biomedical potential. *Livistona chinensis* is a common ornamental plant in Pakistan, also known as the Chinese fan palm, Table palm, or Fountain palm. Its attractive appearance, with large fan-shaped leaves standing on a tall trunk, makes it a popular choice for gardens, parks, and public spaces [19]. Iqbal et al. [20] have utilized this plant to inhibit the growth of both Gram-positive and Gram-negative bacteria. The biochemical compounds present in the extracts of *L. chinensis* are also effective against ROS [21]. Basically, oxidative stress happens when the balance between the oxidants and the defense system of a cell that fights against them gets disrupted [22]. This usually occurs when there is a rise in the levels of these harmful substances like ROS [23,24,25,26]. These harmful substances can cause changes in cellular building blocks such as DNA, proteins, and fats, which could lead to cell death [27]. They can also affect the cell’s membrane and disrupt the functioning of proteins and DNA [28,29]. Because of these changes, oxidative stress has been linked to diseases like cancer, heart problems, and brain disorders [30,31]. To deal with this, antioxidants are needed to counteract these harmful effects [32]. Possessing the ROS scavenging activity makes *L. chinensis* a potential candidate for making AgNPs. In addition to these aspects, AgNPs synthesized from *L. chinensis* are affordable and easy to produce. The main goal of this study was to synthesize AgNPs using a leaf extract from the *L. chinensis* plant (LC-AgNPs) because as a plant it offers a more sustainable and eco-friendly approach compared to traditional methods. Once the nanoparticles were produced, they were characterized using various techniques like UV/VIS spectroscopy, FTIR analysis, zeta potential analysis, XRD analysis, SEM, and metallurgical microscopy. These methods helped us understand different traits of the nanoparticles, such as their size, shape, and the groups of atoms they contain. Understanding the properties of these nanoparticles also contributes to the growing field of nanotechnology, showing us how we can harness the unique characteristics of materials at the nanoscale for various applications in medicine [33]. In addition to this characterization, this study also delved into the possible medical uses of LC-AgNPs by investigating their potential to act as antioxidants, fight against bacteria, and potentially combat cancer. This exploration is important because it uncovers possible new applications for LC-AgNPs that were previously unknown. By understanding and harnessing their unique properties, these nanoparticles could play a significant role in various biomedical fields.

## 2. Results and Discussion

### 2.1. The Preparation of L. chinensis Leaf Extracts

#### 2.1.1. The Evaluation of Different Solvent-Based Leaf Extracts

**Figure 1:**
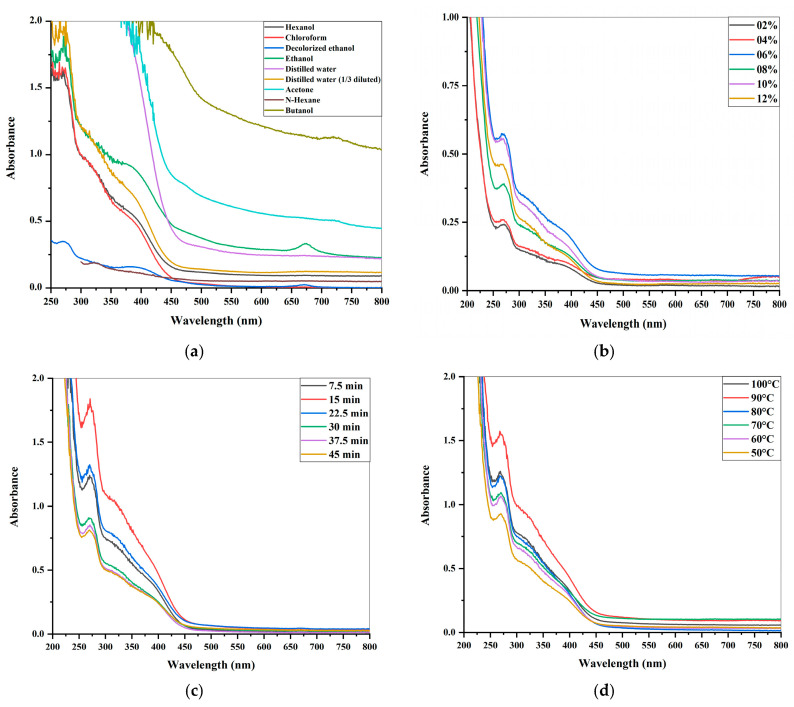
UV–visible spectrums of optimization conditions for efficient leaf extracts: (**a**) different solvents used for leaf extracts, (**b**) different concentrations of leaf extracts, (**c**) dynamic extraction’s effect on leaf extracts, and (**d**) different temperatures for leaf extractions.

The best solvent to obtain an optimal leaf extract was butanol. Similar results are reported by Moradi et al. [34]. They used n-hexane, chloroform, ethyl acetate, and n-butanol to extract pomegranate extract. They found that the highest flavonoid content of 77.1 ± 2.5 mg RUT/g was found in the n-butane fraction, where RUT/g is rutin equivalent/g. This indicates that butanol is a more suitable solvent to obtain the leaf extract of *L. chinensis* than the other tested solvents. The high absorbance value of the butanol-based leaf extracts in Figure 1a indicates that they were efficient enough to provide target compounds of *L. chinensis* in a greater quantity as compared to other organic and inorganic solvents. Following that were acetone, distilled water, ethanol, 10 ppm diluted distilled water, hexanol, chloroform, and treated ethanol-based leaf extracts. Similar conclusions were made in the research of Gali and Bedjou [35]. They reported the comparison of ethyl acetate with butanol-based leaf extracts. The highest phenolic content of 210.00 ± 4.93 µg GAE/mg was shown by the butanol-based leaf extracts, whereas ethyl acetate-based leaf extracts demonstrated a phenolic content of 175.23 ± 5.64 µg GAE/mg.

#### 2.1.2. The Estimation of Variations in Leaf Extract Concentrations

Leaf extracts in distilled water at various concentrations, including 2%, 4%, 6%, 8%, 10%, and 12% were examined. Absorbance of leaf extracts increased by increasing the concentration from 2 to 6%. The 6% leaf extract concentration demonstrated peak maxima at 260 nm. It is illustrated in Figure 1b. This means that by increasing the concentration of leaf powder (solute), more solute is dissolved in the distilled water acting as solvent. Suresh et al. [36] demonstrated that an increase in the concentration of leaf powder from 0.3 to 0.4 g and a further rise from 0.4 g to 0.5 and 0.6 g resulted in an increase in total phenolic and flavonoid contents. An extreme decline was observed at an 8% concentration. It could be due to the fact that at a 6% leaf extract concentration, the leaf powder reaches its maximum solubility with distilled water and becomes saturated. However, further increases in leaf concentration may lead to aggregation. Moreover, up to a 6% concentration of leaf extract, the number of absorbing molecules goes on increasing and results in higher absorbance.

#### 2.1.3. Dynamic Extraction’s Effect on Leaf Extract

The effect of time on 6% leaf extract was investigated. For this purpose, leaf extracts were observed every 7.5 min for a total period of 45 min. The 6% concentration of leaf extracts initially exhibited an increase in absorbance by increasing the time period from 7.5 to 15 min. The further increase in time affected the absorbance negatively. The yield of leaf extracts tended to increase when the extraction time increased. However, with a prolonged time, the risk of thermal degradation of the polyphenol chemical structures was inevitable [37]. Peak maxima were observed at 260 nm after 15 min and a 45 min time period revealed the lowest peak. There is a time limit for each of the bioactive compounds present in the matrix in which quality yields can be obtained. For this reason, the extraction time can vary from a few minutes to several hours in some cases [38]. Figure 1c shows that in this case, compounds reacted to give products in the first 15 min. After this time, no more reaction occurred due to the unavailability of solvent. In addition, the chemical and enzymatic reactions break down the compounds in the leaf extract as the time exceeds 15 min. The resulting degradation process led to the loss of functional groups alongside the formation of byproducts. It was indicated by a continuous decrease in absorbance from 22.5 to 45 min in spectra. Similar results were reported when Yerena-Prieto et al. [39] optimized the extraction time of *Moringa oleífera* Lam. using an ultrasonic-assisted extraction method. The extraction time from 2 to 30 min was evaluated and 15 min was found to be the optimal time.

#### 2.1.4. The Influence of Temperature on Leaf Extract

The leaf extracts at a 6% concentration were utilized to estimate the effect of temperature. The samples in six different flasks were tested at 100, 90, 80, 70, 60, and 50 °C. UV/VIS spectroscopy was performed. In the beginning, by increasing the temperature from 50 to 90 °C, the absorbance increased efficiently. Peak maxima were observed at 90 °C at the wavelength of 260 nm and depicted in Figure 1d. The increase could be due to increased reactivity or greater solubility of *L. chinensis* compounds. As the temperature increased above 90 °C, some compounds in *L. chinensis* may have lost their absorbing property, started to degrade, or chemically changed. Some heat-sensitive compounds and proteins in the plant extract also structurally changed or underwent denaturation which resulted in a drastic decline in the peak at 100 °C. Although high temperature favored the discharge of phenolic compounds, due to their thermal instability, phenolic compounds were degraded at a high temperature and pressure. For instance, Pimentel-Moral et al. [40] studied *Hibiscus sabdariffa* to extract its phenolic compounds and observed degradation of some of its thermo-labile compounds above 100 °C.

Okiyama et al. [41] also reported a decrease in total flavonol content after 40 min of extraction at 75 to 90 °C. This means that the extraction of phenols and flavonoids was increased by increasing temperature but up to a certain level. After that certain limit, the degradation of compounds starts. This might be the case with *L chinensis* extract where after 90 °C, a sudden drop in absorbance was evident and could be attributed to a degradation of compounds. Ersan et al. [42] reported a similar behavior in the phenolic compounds of *pistachio hulls* at higher temperature. When the temperature for extraction was increased from 110 to 150 °C, the phenolic yield increased. However, a further increase in temperature from 170 to 190 °C led to a significant decrease in the yield of phenolic content.

### 2.2. The Metallurgical Microscopy of L. chinensis Leaf Particles

**Figure 2:**
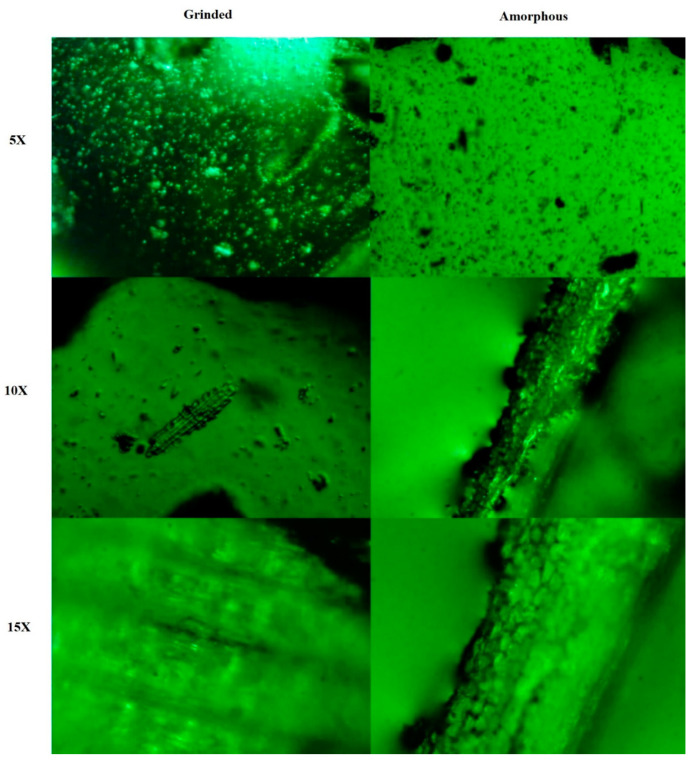
Metallurgical microscopy of *L. chinensis* leaf particles.

Metallurgical microscopy is a novel and extremely effective approach to pattern recognition in optical images for advanced materials characterization. In the present study, metallurgical microscopy was performed on *L. chinensis* leaves. The plant leaves were studied in finely ground and powdered/amorphous form. Metallurgical microscopy was accomplished at 5X, 10X and 20X magnifications. The findings of the microscopy provided insights into the structural characteristics of the leaf at different magnifications, shedding light on the microscopic features and behaviors under examination. The first image shows fine granular particles at 5X resolution. Further magnification at 10X and 15X increased the visibility of the structures. The particles showed irregular shapes arranged in tubular forms. In a similar study, Link et al. [43] have shown the ash melting behavior of the blend of reed and wheat straw to be a complex process. The study involved metallurgical microscopy. From the results, it was deduced that ash melting could cause severe problems in boiler operation, such as agglomeration of the fluidized bed. There is a clear demarcation of finely ground and amorphous leaves in Figure 2.

### 2.3. The UV/VIS Analysis of Plant Extract and LC-AgNPs

**Figure 3:**
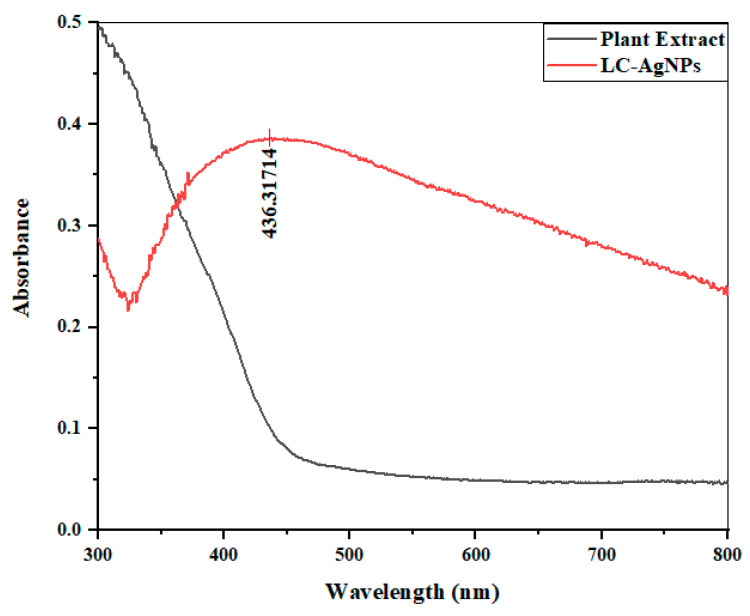
UV/VIS spectroscopy of plant extract and LC-AgNPs in comparison.

UV/VIS spectroscopy of *L. chinensis* leaf extract and the reaction mixture was performed to confirm the fabrication of LC-AgNPs. When silver nanoparticles are exposed to UV/VIS light, the conduction electrons on the nanoparticle’s surface undergo collective oscillations. This oscillation is what we call localized surface plasmon resonance (SPR). The frequency of the incident light matches the natural frequency of the electron oscillations, satisfying the resonance condition. This resonance leads to a strong absorption and scattering of light by the nanoparticles [44]. In this study, no absorbance peak was visible in the plant extract. However, a clear SPR band at 436.31 nm affirmed the formation of LC-AgNPs in the reaction mixture, as the SPR band between 410 and 450 nm is characteristic of silver nanoparticles as shown in Figure 3. The outcomes were quite similar in nature when researchers utilized extracts obtained from a variety of plant sources including *Origanum majorana*, *Dovyalis caffra*, *Persicaria odorata*, *Hypericum perforatum*, *Juniperus procera*, and *Erythrina abyssinica* for the purpose of producing silver (Ag) nanoparticles. The results obtained from these different plant extracts yielded comparable findings, suggesting a commonality in their potential to facilitate the production of Ag nanoparticles [45,46,47].

### 2.4. FTIR Analysis

**Figure 4:**
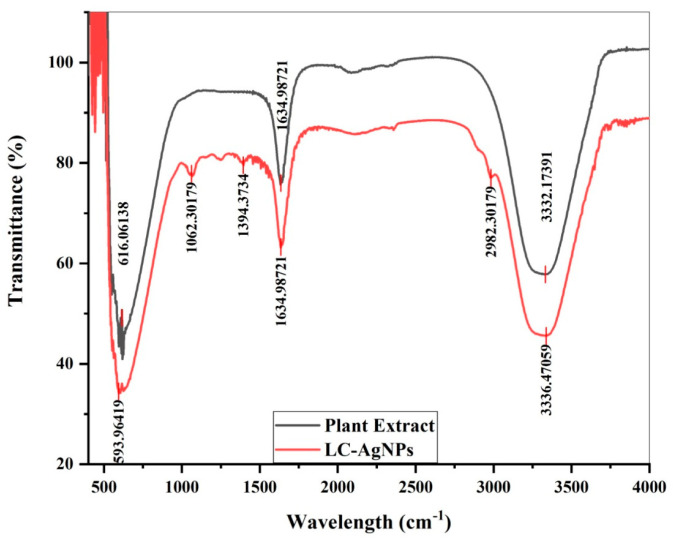
FTIR spectroscopy of LC-AgNPs and the plant extract.

*L. chinensis* leaf extracts and LC-AgNPs underwent FTIR analysis. FTIR spectra against each sample were drawn using the software Origin95. The FTIR spectra of immobilized LC-AgNPs and plant extract are shown in Figure 4. Peaks of LC-AgNPs were located at 593.96, 1062.30, 1394.37, 1634.98, 2982.30, and 3336.47 cm^−1^. The peaks were assigned to specific biomolecules. The peak at 595.19 cm^−1^ indicated the bonding of the C–C–C–N group of nitrites [48]. Similarly, the C–H bond stretching of cellulose was depicted by the peak at 1062.30 cm^−1^ [49]. The peaks at 1394.37, 1634.98, and 2982.30 cm^−1^ indicated the –C–H– bending, C=O stretching, and C–H stretching of polyphenols, ketones, and alkanes [50,51,52]. Finally, the last peak at 3345.67 cm^−1^ showed the C=C stretching of alkenes [53]. The peaks highlighted the presence of nitrites, alkanes, alkenes, and proteins in LC-AgNPs.

Contrary to the immobilized AgNPs, the plant extract depicted only three peaks at 616.06, 1634.98 and 3332.17 cm^−1^. The peak at 616.06 cm^−1^ indicated the presence of C–Cl stretching of alkyl groups [54]. The OH stretching of ketones is depicted by the peak at 1634.98 cm^−1^ [55]. The third and last peak at 3332.17 cm^−1^ highlighted the presence of OH stretching of phenolic compounds [56]. It was noticed that the peak at 3345.67 cm^−1^ was common in all of these samples. LC-AgNPs and free AgNPs showed six peaks, whereas only three peaks were observed in the spectra showing leaf extract FTIR results. Table 1 compares the values obtained from the FTIR analysis with the literature to show the functional groups present in LC-AgNPs, free AgNPs, and the plant extract. In short, it can be concluded from the FTIR analysis that the presence of ketones and phenolic compounds have played a specific role in the formation of AgNPs. Both of these compounds act as reducing agents and convert the Ag^+^ into Ag^0^ by donating electrons. Both can also stabilize the formed AgNPs. They get adsorbed onto the surface of nanoparticles, prevent agglomeration, and enhance the stability of the colloidal solution [57]. Oves et al. [54] also reported the role of phenolic and alcoholic compounds in the formation of AgNPs when they used the extract of *Conocarpus lancifolius*.

### 2.5. XRD

**Figure 5:**
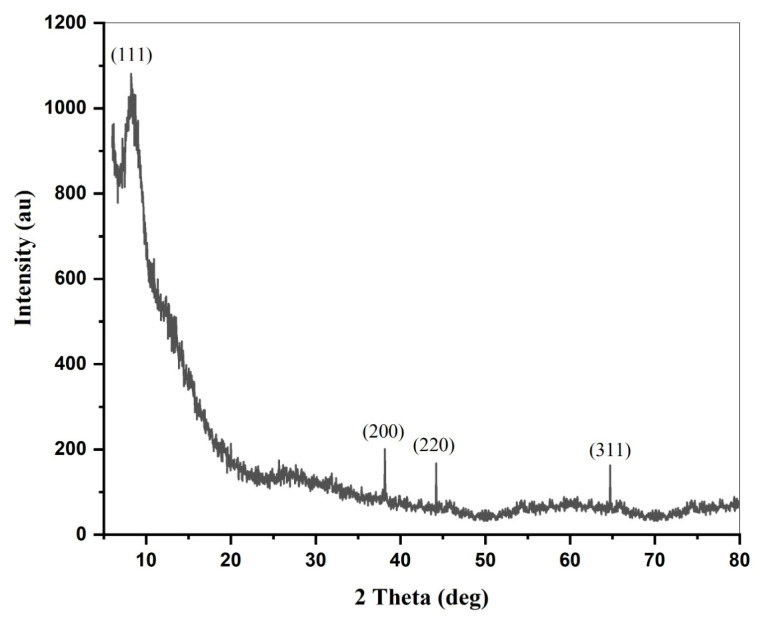
X-Ray diffraction analysis of LC-AgNPs.

LC-AgNPs were subjected to X-Ray diffraction analysis. Figure 5 demonstrates the XRD profile of the LC-AgNPs. Spectra were drawn using the Origin 95 software. The X’Pert high score was used to spot the index of these peaks and compared with the standard values from the JCPDS. The peaks corresponding to 2θ were observed at 8.24, 38.16, 44.20, and 64.72°. The hkl planes of these peaks were (111), (200), (220), and (311) respectively. Manikandan et al. [58] used the leaf extract of *Ocimum americanum* (Hoary Basil) for the production of AgNPs and found angles corresponding to the (111), (200), (220), and (311) planes, which showed the successful formation of AgNPs. In another study, Pungle et al. [59] used the *Tridax procumbens* extract for the production of AgNPs. They carried out XRD analysis for viewing the crystalline nature of AgNPs. The angles found corresponded to the (111), (200), (220), and (311) planes, which indicated the face-centered cubic structure of AgNPs. The results also correlate with those of Shyamalagowri et al. [60], where AgNPs were produced using an extract of *Hylocereus undatus*. They also found angles corresponding to the (111), (200), (220), and (311) planes, which depicted the formation of FCC AgNPs. Comparing these results with ours, it can be concluded that the LC-AgNPs formed face-centered cubic structures in nature.

### 2.6. Zeta Potential

**Figure 6:**
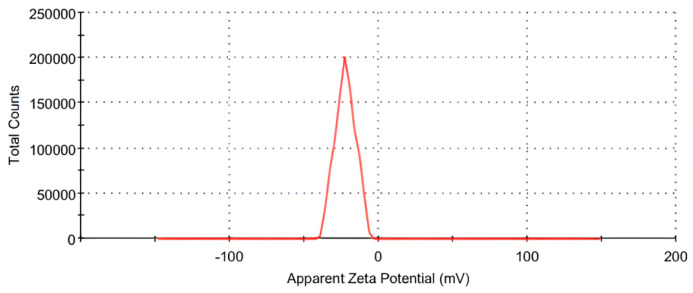
Zeta potential distribution of LC-AgNPs.

Zeta potential analysis was conducted to estimate the stability of LC-AgNPs [61]. Zeta potential analysis provided information about the stability and surface charge of colloidal LC-AgNPs. The system was set at 25 °C for 12 zeta runs. The designed count rate was 253.3 kcps. However, measurement position was 2 mm. Only a single peak appeared with a mean value of −22.3 mV. The calculated standard deviation was 6.79 mV. The mean value was the zeta potential of the sample. Zeta deviation was 6.79 mV with a conductivity of 0.0206 mS/cm. The cell description consisted of clear disposable zeta cells and the result quality was good. The occurrence of a sharp peak at −22.3 mV indicated that AgNPs are negatively charged and dispersed in the medium (Figure 6). The presence of negative charge showed the repulsion between particles and suggested that AgNPs are very stable in nature [62]. The results were consistent with the behavior of other plants that have been employed for the production of AgNPs. The zeta potential of −22.3 mV was also reported by Bharadwaj et al. [61] when they used the *Diospyros malabarica* fruit extract for the production of AgNPs. In another study, AgNPs were also produced by using *Melia azedarach*. They were also found to be stable and well dispersed in the medium as they showed the zeta potential of −22.3 mV [63]. Chandraker et al. [64] also found the zeta potential of AgNPs to be −22.3 mV when they used an extract of *Rubia cordifolia*. Hence, the results of this research about LC-AgNPs are consistent with the studies indicating the AgNPs are well dispersed and stable.

### 2.7. LC-AgNPs Size Distribution by Intensity

**Figure 7:**
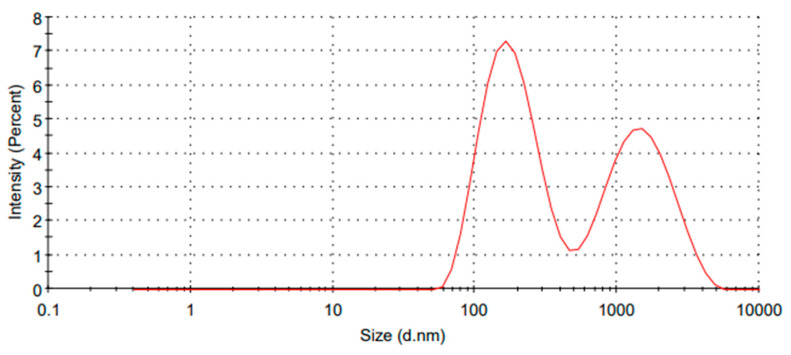
Size distribution by intensity of LC-AgNPs.

Zetasizer was utilized to find the size distribution of AgNPs. Therefore, the size distribution of LC-AgNPs was also estimated by zetasizer [58]. The temperature was set at 25 °C and a 70 s duration was used. The count rate was 197.4 kcps with a measurement position adjustment at 5.50 mm. Two peaks of size 189.4 and 1564 d.nm were observed with a percentage intensity of 56.2 and 43.8, respectively. Peak 1 showed a standard deviation of 85.06 d.nm, whereas a standard deviation of 809.0 d.nm was shown by peak 2. The calculated z-average was 255.7 d.nm. The polydispersity index (pdI) was 0.463. The cell description was a clear disposable zeta cell. The Z average diameter of AgNPs was found to be 255.7 nm and illustrated in Figure 7. Tormena et al. [65] used *Handroanthus impetiginosus* for green synthesis of AgNPs. They performed zetasizer analysis and found a peak at 255 nm which correlates with our results.

The presence of two peaks indicated the formation of different sizes of particles. However, the peak at 1564 nm can be attributed to the aggregation of AgNPs in the sample [66]. Therefore, average size was estimated to be close to 189.4 nm, which was 255.7 nm. However, this larger size was attributed to the aggregation of particles in the solution. A similar size distribution was also indicated by Thirumagal et al. [67] who used the *Justicia adhatoda* L. leaf extract for AgNPs synthesis. The results also correlate with Jyoti et al. [68], who produced the AgNPs from the leaf extracts of *Picrasma quassioides*.

### 2.8. SEM

**Figure 8:**
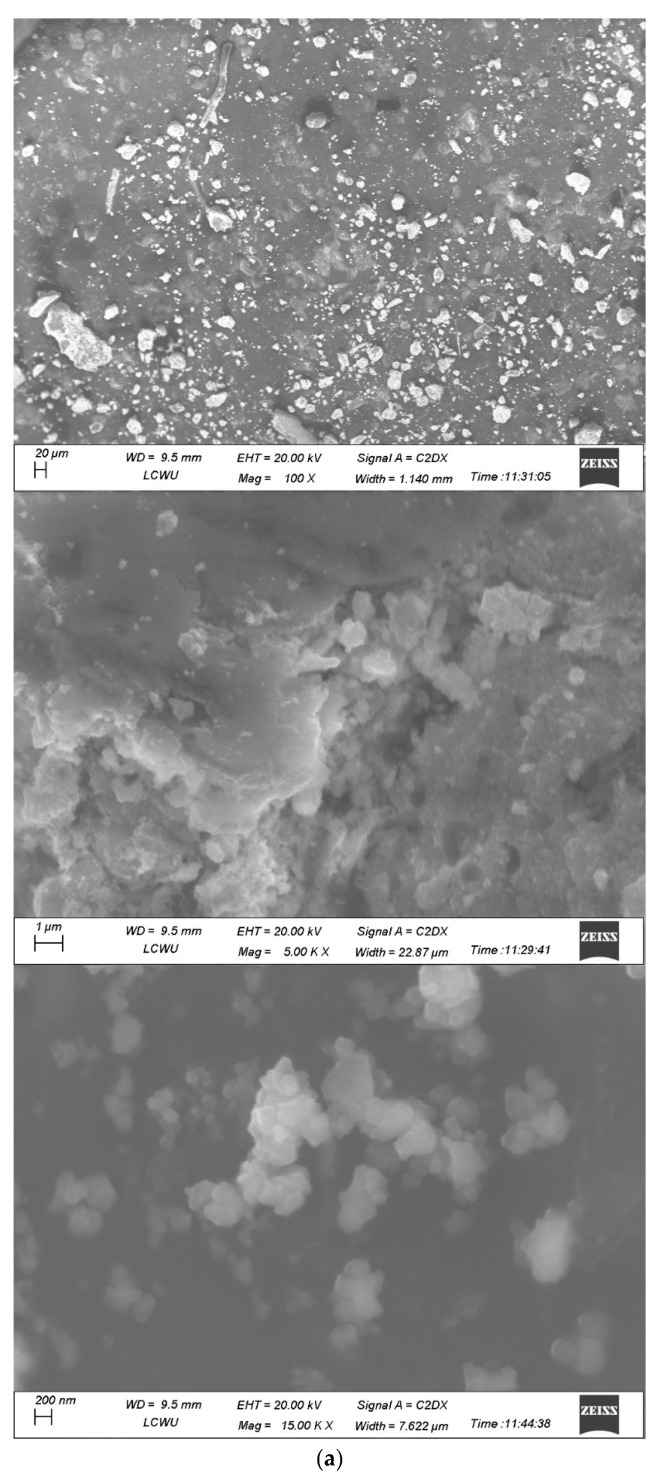

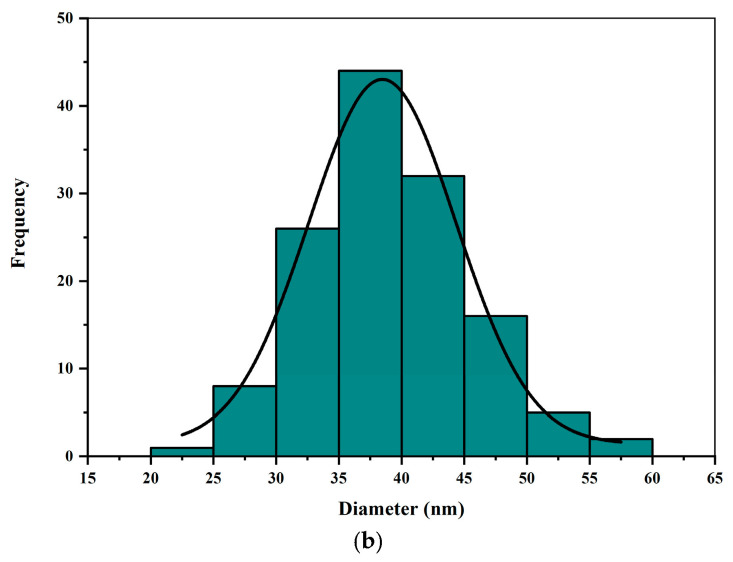
(**a**) SEM images of LC-AgNPs (**b**) Size distribution histogram of LC-AgNPs.

The SEM images of LC-AgNPs were illustrated at 100X, 5000X and 15,000X in Figure 8a. The images showed the irregular shape of LC-AgNPs with some dominant spherical LC-AgNPs. ImageJ software was utilized for measuring the sizes of LC-AgNPs from the image at 15,000X magnification. The software calculated the size of 134 LC-AgNPs. The size gave an estimation for the area of these LC-AgNPs. The area was utilized to calculate the diameter. The highest number of nanoparticles were present in the range of a 30–45 nm diameter. The same size distribution has been reported by Ghabban et al. [69]. Moreover, Sana and Dogiparthi, [70] also reported the size distribution of 30–40 nm when producing AgNPs using the leaf extracts of *Givotia moluccana*. The AgNPs produced by Rajendran et al. [71] using the extract of *Origanum heracleoticum* also ranged between 30 and 40 nm in size.

A histogram of the mean average size of LC-AgNPs was drawn. The histogram was plotted using the Origin95 software. The maximum size was calculated to be between 30 and 40 nm. A higher size distribution was evident between 30 and 50 nm and the average mean size of LC-AgNPs was found to be 38.46 ± 0.26 nm. Gaussian law was applied to find the mean average size. The histogram reveals the size distribution of LC-AgNPs in Figure 8b. The AgNPs produced from the extracts of *Holoptelea integrifolia*, *Eugenia roxburghii*, and *Millettia pinnata* also had the mean size of 38 nm [62,72,73].

### 2.9. Antioxidant Activity

**Figure 9:**
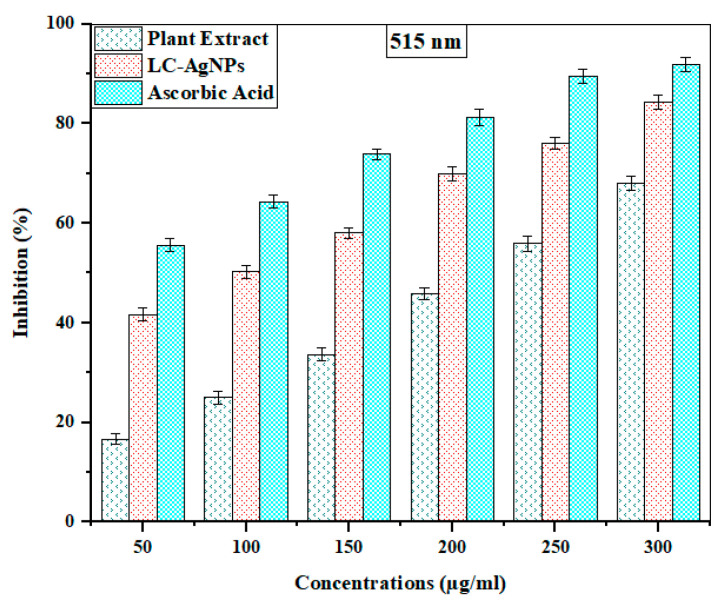
Bar graph depicting DPPH radical scavenging activity of *L. chinensis* extract, LC- AgNPs, and ascorbic acid.

The synthesized LC-AgNPs were assessed for their antioxidant activity by performing the DPPH assay. In the DPPH assay, DPPH• is a free radical that is purple in color. This free radical can be reduced by hydrogen donors like antioxidants. This gives a purple color at 515 nm and after reduction it starts to shift its color to pale yellow. The higher the amount of antioxidants in the compounds, the more pale yellow color is formed [74]. In the current study, the DPPH scavenging activity of *L. chinensis* extract and LC-AgNPs was investigated with ascorbic acid acting as the standard. Various concentrations (50, 100, 150, 200, 250, and 300 µg/mL) of *L. chinensis* leaf extracts, LC-AgNPs and ascorbic acid were allowed to react with a 0.002% solution of DPPH in the dark. The absorbance was examined at 515 nm. The percentage inhibition was calculated by putting the absorbance values in the formula. The ascorbic acid, plant extract, and LC-AgNPs demonstrated dose-dependent behavior. Increases in concentration resulted in an increase in the percentage radical scavenging activity of all samples. Kumar et al. [75] also found dose-dependent behavior for AgNPs when performing the DPPH assay. At 515 nm, ascorbic acid showed a 91.84 ± 1.47% scavenging activity as the highest value. The maximum DPPH scavenging activity of LC-AgNPs was 84.24 ± 1.43%. However, the plant extract demonstrated the highest radical scavenging activity of 68.00 ± 1.39% at this wavelength. Dose-dependent behavior was seen by the absorbance values at 515 nm. However, it was found that AgNPs had higher ROS scavenging activity compared to the plant extract but it had lower antioxidant activity compared to ascorbic acid. An IC_50_ of 85.01 ± 0.17% was exhibited by AgNPs (shown in Table 2). This value was good compared to the IC_50_ values obtained from the AgNPs synthesized from *S. officinalis* [76] and this indicated that AgNPs could be employed for the production of antioxidant drugs. The bar graph in Figure 9 also shows these results.

Similar results for the IC_50_ were obtained when Tyagi et al. [77] used a *Tagetes erecta* extract for the production of AgNPs and evaluated their antioxidant activity. They reported the same results, where the pattern of antioxidant activity was ascorbic acid > AgNPs > plant extract. Fierascu et al. [78] found the antioxidant activity of plant extract, ascorbic acid, and AgNPs synthesized from *Rosmarinus officinalis* and found that AgNPs were more effective than the plant extract. Other studies performed by Rajakannu et al. [79] and Alshmgani et al. [80] also came to the similar conclusion that AgNPs were more effective than *Catharanthus roseus* and *Garcinia mangostana* extracts, indicating the higher antioxidant potential of AgNPs.

### 2.10. Antibacterial Activity

**Figure 10:**
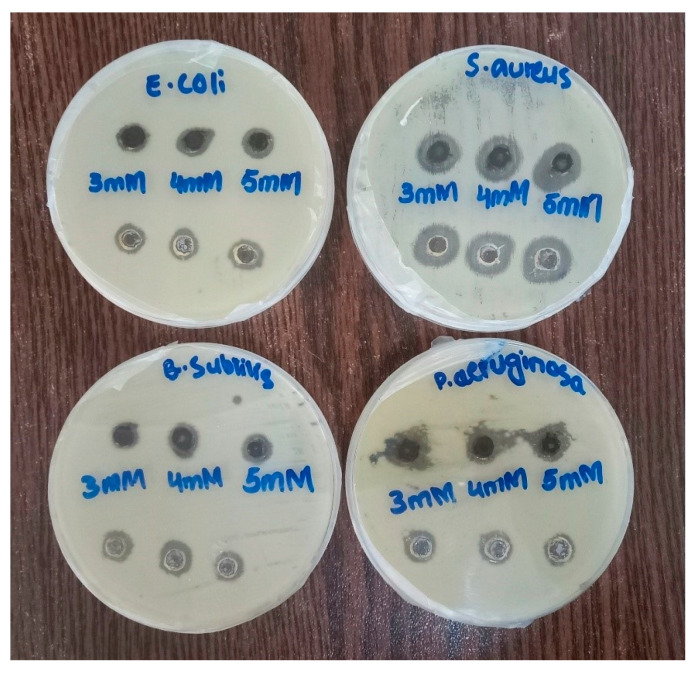
Agar well diffusion assay against *E. coli*, *S. aureus*, *B. subtilis*, and *P. aeruginosa* after 24 h.

The antibacterial potential of LC-AgNPs was investigated and compared with that of free AgNPs. Antibacterial activity of LC-AgNPs and free AgNPs was found against two Gram-positive and two Gram-negative bacteria. The Gram-positive bacteria included *S. aureus* and *B. subtilis* while the Gram-negative bacteria included *P. aeruginosa* and *E. coli*. The antimicrobial activity of LC-AgNPs was estimated by using free AgNPs as a control. Three different concentrations (3, 4 and 5 mM) of LC-AgNPs and free AgNPs were used. The antibacterial activity was tested after 24 h. An agar well diffusion assay was performed and zones of inhibition were recorded as shown in Figure 10. After 24 h, free AgNPs (4 and 5 mM) showed a maximum of a 15 ± 0.5 mm diameter zone of inhibition against *S. aureus.* Similar results were depicted by Qais et al. [81] when they used AgNPs against resistant *S. aureus*. In the case of LC-AgNPs, 4 and 5 mM concentrations were effective enough to demonstrate a maximum of a 16 ± 0.8 mm diameter zone of inhibition against *P. aeruginosa*. Younas et al. [82] also found that, of the Gram-negative bacteria, the maximum zone of inhibition for AgNPs was against the *P. aeruginosa*. Free AgNPs of 3 mM concentration demonstrated a minimum of a 8.6 ± 0.5 mm diameter zone of inhibition against *E. coli*, which might be attributed to the outer membrane providing some level of protection against antimicrobial agents [83]. However, 3 and 5 mM concentrations of LC-AgNPs demonstrated the lowest zone of inhibition (9 ± 0.2 mm diameter) against *B. subtilis*. Table 3 shows the complete data for antibacterial activity.

There are certain reasons for the antibacterial activity of the AgNPs. The AgNPs release silver ions that gain entry into the cell through pores. After gaining entry they can disrupt DNA replication in the cell [84]. Loss of permeability, ROS generation, or the direct damage to the cell membrane of bacteria also contribute to cell death [85]. The actual mechanism involved in the antibacterial potential is still unknown. However, solid evidences support that AgNPs release silver ions which can interact with the DNA or RNA or nucleosides of these nucleic acids [86]. Silver ions also have an affinity for the sulfur proteins which help them in binding with cytoplasm or the cell wall. These mechanisms interfere in ATP synthesis and also have an effect on ROS production. In addition, silver ions can also cause denaturation of ribosomal components and can stop protein synthesis [3].

### 2.11. Anticancer Activity

The MTT assay was employed for assessment of anticancer activity. Its principle is simple, as where the cell lines are reacting with the samples, MTT dye is added. The enzymes of active, or live cells, react with it to form formazan crystals of purple color [87]. Then dimethyl sulfoxide (DMSO) is added to dissolve the crystals and the color change is observed. The higher the number of live cells, the more color there is and the sample is less effective [54]. Therefore, the anti-proliferative effects of the plant extract, free AgNPs, and LC-AgNPs were investigated in human thyroid cancer cells (TPC1 cell line) using the MTT assay. In vitro cell cycle analysis of TPC1 cells was conducted by treating them with *L. chinensis* extract, free AgNPs, and LC-AgNPs. TPC1 had 88 cells viability before loading the samples into them and this was referred to as the control. After this, samples were diluted 10% and 15% in phosphate buffer saline (PBS) and the effect was observed after 30 and 60 min in both cell lines. Here, the dilution refers to 10% and 15% samples in 90% and 85% of PBS solution. Samples were diluted only 5% to investigate the results after a 90 min exposure. The optimum results were exhibited by LC-AgNPs with 15% dilution after 30 min in the TPC1 cell line. This was followed by the plant extract as well as free AgNPs. In TPC1, the 15% diluted sample of LC-AgNPs showed 38% cell viability in a 30 min exposure. This was the optimal anticancer activity in all samples. The plant extract and free AgNPs exhibited 45% and 56% cell viability under the same conditions.

**Figure 11:**
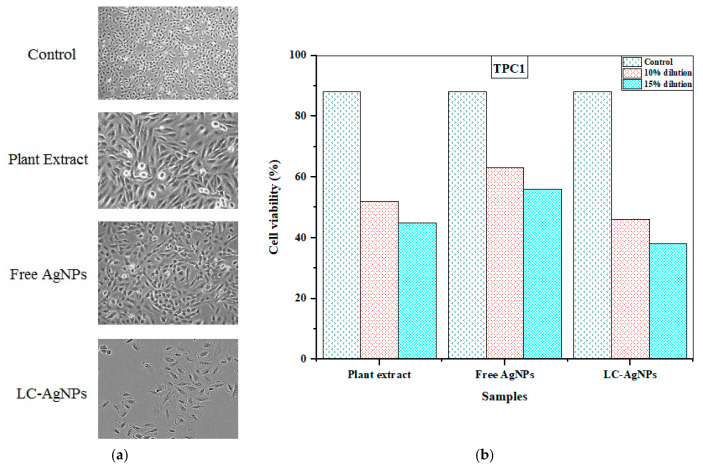
(**a**) TPC1 cell lines under the influence of plant extract, free AgNPs, and LC-AgNPs. (**b**) Bar graph depicting 10 and 15% dilutions of plant extract, free AgNPs, and LC-AgNPs in comparison with control during 30 min exposure in a TPC1 cell line.

The 90 min exposure of each sample was the least effective in the cell line as the resulting values were very close to the control. When samples were diluted to 5%, the plant extract, free AgNPs and LC-AgNPs demonstrated 76, 82 and 70% cell viability. This is demonstrated in Table 4. Saber et al. [88] observed a similar result for AgNPs when synthesized from *Trapa natans* extract and tested against A431 human skin cancer cells. The results of this study align with the results of others, where AgNPs were synthesized from the extracts of *Dodonaea viscosa* [89], *Hypericum perforatum* L. [90], *Azadirachta indica* [91], and *Tamarindus indica* [92] and tested against cancer cell lines. The results are demonstrated in pictorial form in Figure 11a. It was seen that when TPC1 cell lines encountered the plant extract, the number of cells decreased. The same happened with the free AgNPs, but when LC-AgNPs were used the number of cells decreased drastically signifying a high anticancer activity. The bar graph in Figure 11b shows the same results. The exact mechanism of the anticancer activity of LC-AgNPs is unknown. However, the FTIR analysis suggested the presence of phenolic compounds as capping agents in the LC-AgNPs and they have been reported to induce apoptosis, or programmed cell death, in cancer cells [93]. According to El Raey et al. [94], apoptosis was induced by the phenolic compounds present as capping agents in the AgNPs. Similarly, many studies have suggested the possible role of biochemical compounds in the anticancer activity [95,96]. The precise mechanism underlying the anticancer activity of LC-AgNPs remains unidentified. Nevertheless, there are various factors that can be considered to elucidate the reduction in the number of cancer cells. Datkhile et al. [97] suggested the potential reason for this phenomenon could be attributed to the excessive generation of reactive oxygen species (ROS). ROS can lead to DNA and mitochondrial damage, ultimately causing cell death. In another study, Lydia et al. [98] used AgNPs produced from *Carica papaya* against MCF-7 and Hep-1 cell lines and found that tannins and flavonoids could also be responsible for antitumor activities, suggesting the role of biochemical compounds. However, these studies suggested that various mechanisms could be responsible for the anticancer activity of LC-AgNPs. These finding suggested that LC-AgNPs possess an anticancer activity greater than that of plants and free AgNPs, which should be exploited to develop drugs against cancer. However, further studies are required in this field to explore the exact mechanisms behind their action.

## 3. Materials and Methods

### 3.1. Chemicals and Reagents

Silver nitrate (AgNO_3_), methanol (CH_3_OH), nutrient agar, potassium persulfate (K_2_S_2_O_8_), DPPH (2,2-diphenyl 1 picrylhydrazyl), ABTS {2,2′-azino-bis(3-ethylbenzothiazoline-6-sulfonic acid) diammonium salt}, and Luria-Bertani (LB) medium-containing agar were provided by Sigma-Aldrich Chemical Co., (3050 Spruce St., Saint Louis, MO, USA, 63103). RPMI 1640 (Roswell Park Memorial Institute), streptomycin, and amphotericine B (Gibco, Paisely, UK) were also obtained.

### 3.2. The Collection and Pre-Treatment of Livistona Chinensis Leaves

The fresh leaves of *L. chinensis* were acquired from the Botanic Garden of GC University, Lahore. The leaves were properly cleansed with faucet water to wipe off dust and other contaminants, and air-dried for 3 h. After that, they were cut into small pieces. The pieces were shade-dried completely for 4 days to avoid direct sunlight exposure.

### 3.3. Preparation and Parameter Optimization for Efficient Leaf Extracts

The fully dried leaves were ground into fine powder. The extracting solvent, leaf powder concentration, extraction time and temperature were optimized to obtain the finest leaf extracts. Leaf powder (5 g) was added to each flask containing 50 mL of n-butanol, hexanol, chloroform, acetone, n-hexane, ethanol, and distilled water. The extraction was performed at 80 °C in a shaking water bath at 120 rpm for 15 min. The aqueous extract was subjected to centrifugation (4000 rpm, 15 min) using a centrifuge (Sigma Laboratory Centrifuges 3K30, Newtown, Wem Shropshire, UK). The supernatant was stored, whereas organic solvents underwent lyophilization. Leaf concentrations were optimized by making a 2–12% solution in distilled water and placed in a shaking water bath at 80 °C for 15 min. After obtaining the best weight of the leaf powder, a leaf extract was obtained by utilizing the optimum concentrations after 7.5, 15, 22.5, 30, 37.5, and 45 min, keeping all the other conditions the same as before. The temperatures, such as 50, 60, 70, 80, 90, and 100 °C, were systematically scrutinized to find the most suitable temperature for leaf extraction while maintaining all conditions optimized. All the filtrates were sent for UV–visible spectrophotometry in the Nanotechnology Lab, Department of Chemistry, GC University Lahore.

### 3.4. The Lyophilization of Organic Filtrates

The filtrates of all organic solvents, except chloroform and hexanol, were lyophilized at −52 °C for 10 days (9 h a day) using a benchtop lyophilizer (Martin Christ Alpha 1-4 LD, An der Unteren Söse 50 37520 Osterode am Harz Germany). The fine powders of these filtrates were diluted again in 50 mL of distilled water. Chloroform and hexanol were absorbed by the leaves during the extraction process, so 25 mL of distilled water was added, and then centrifugation was carried out at the aforesaid conditions. The supernatant was taken and liquefied further with distilled water to raise the total volume up to 50 mL.

### 3.5. The Metallurgical Microscopy of L. chinensis

The leaves of *L. chinensis* were subjected to grinding to obtain them both in ground and powdered form. The samples were further prepared by adding glycerol to the leaf powder and this made a homogenous mixture with an even surface. At that point, these samples were sent to the Center for Advanced Studies in Physics (CASP), GC University Lahore, for metallurgical microscopy. The settings for metallurgical microscopy resolutions were adjusted to 5, 10, and 20X.

### 3.6. The Green Biosynthesis of AgNPs

**Figure 12:**
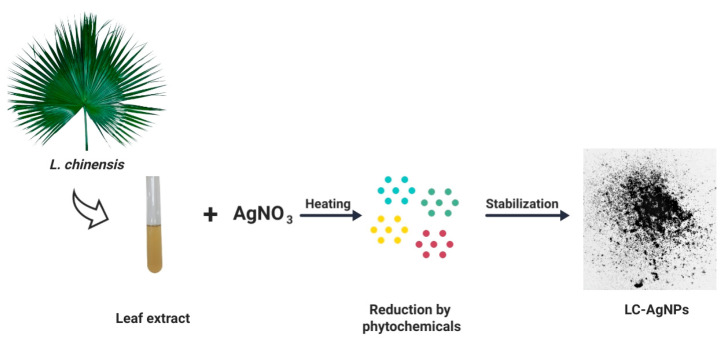
Synthesis of LC-AgNPs by using leaf extracts of *L. chinensis*.

The AgNPs were bio-fabricated from the leaf extracts of the fountain palm (LC-AgNPs) by following the approach used by Manikandan et al. [58]. Leaf extract (2 mL) was added into 8 mL of 4 mM AgNO_3_ and kept for 90 min at 70 °C and 120 rpm in a shaking water bath. The color change from yellow to dark brown indicated the formation of LC-AgNPs. In Figure 12, the flowsheet of AgNPs synthesis from *L. chinensis* extract is depicted.

### 3.7. Pellet Formation

The green synthesized nanoparticles were subjected to centrifugation using a centrifuge. The centrifugation was accomplished at 4000 rpm for 20 min to obtain the pellet. The supernatant was discarded, and the pellet was washed twice with distilled water and once with ethanol. The pellet was stored overnight in the dark at 25 °C to evaporate the ethanol. The dried pellet was crushed into fine powder using a pestle and mortar, and stored at 4 °C for further investigation.

### 3.8. The Production of Free AgNPs

Free AgNPs were prepared using the same method that was used for LC-AgNP synthesis. The only difference is that the leaf extract was substituted with distilled water.

### 3.9. The Characterization of LC-AgNPs via UV/VIS, FTIR and XRD Analysis

For the sake of primary confirmation, the sample prepared under the aforesaid optimized conditions was diluted with distilled water to achieve a 10 ppm concentration and observed at the 200–800 nm wavelength range for UV/VIS spectroscopy [99]. For functional group evaluation, liquid samples of LC-AgNPs and plant extract were sent to the CASP GC University Lahore for FTIR analysis. The spectra were documented on an FTIR spectroscope (Spectrum-100, Perkin-Elmer, St. Louis, MO, USA). The percentage transmittance was evaluated at a range of 4000–400 cm^−1^ and 25 °C [100]. The liquid sample of LC-AgNPs was also sent to the Central Research Lab, Lahore College for Women University (LCWU), Lahore for XRD analysis to estimate the crystalline nature. An XRD machine (D8 discover, Bruker Billerica, MA, USA) in the 2θ range 5° to 80° with a scan speed of 1 determined the crystalline nature of the LC-AgNPs [101]. This produced a Kα of wavelength 0.15406 nm utilizing a copper anode.

### 3.10. Zeta Potential Measurement

The powdered sample of AgNPs was conveyed to the Syed Babar Ali School of Science and Engineering, Lahore University of Management Sciences (LUMS) for zeta potential analysis. The sample underwent sonication and was examined using Zetasizer Nano ZS instruments (Zetasizer Ver. 7.10, Malvern Instruments Ltd., Worcestershire, UK). The examination was employed at room temperature with a count rate of 253.3 kcps and in the range of −150 mV to 150 mV apparent zeta potential [102]. Moreover, the size distribution of particles was also found using the zeta sizer. The count rate for size distribution by intensity of LC-AgNPs was 197.4 kcps at 25 °C. The measurement position was 5.50 mm with 255.7 d. nm Z-average.

### 3.11. SEM

SEM was executed to estimate the size distribution and morphology. A powdered sample of LC-AgNPs was consigned to the Central Research Lab, LCWU, for SEM (EVOLS10, ZEISS Co., Jena, Germany) analysis. The size distribution of biogenic AgNPs was observed [103].

### 3.12. Biomedical Applications

#### 3.12.1. Antioxidant Activity via DPPH Assay

The antioxidant potential of synthesized LC-AgNPs was determined employing 2,2-diphenyl-1-picrylhydrazyl (DPPH). The procedure outlined by Kharat and Mendhulkar [104] was implemented with slight variations. The twenty microliters of different concentrations (50, 100, 150, 200, 250, and 300 µg/mL) of LC-AgNPs, plant extract, and ascorbic acid were added into 180 µL of 0.002% DPPH solution in methanol to make the total volume 200 µL in Eppendorf tubes. These Eppendorf tubes were subjected to vortex mixing and then 2.8 mL of distilled water was added. Consequently, they were incubated in the cold cabinet (MPR-1410, SANYO, Japan) at 4 °C for 30 min. The DPPH solution (0.002%) was used as the control, and the absorbance was observed at 515 nm using a spectrophotometer (Model: UV—1700, Shimadzu Corporation, Kyoto, Japan). The percentage scavenging activity was computed by applying the following formula:

$$\mathrm{Percentage}\;\mathrm{scavenging}\;\mathrm{activity}=\frac{Ac-As}{Ac}\times 100$$

where “*Ac*” is absorbance of control and “*As*” is the absorbance of the sample, respectively.

#### 3.12.2. Antibacterial Activity

The antibacterial potential of the synthesized LC-AgNPs and free AgNPs was examined against *E. coli*, *B. subtilis*, *P. aeruginosa*, and *S. aureus.* These cultures were collected from the culture bank of GC University, Lahore, and were refreshed before use. Nutrient agar was prepared and sterilized using an autoclave (Model: WAC-60, Wisd, WiseStri, Germany). The agar well diffusion method of Pungle et al. [105] was performed against different concentrations encompassing 3, 4, and 5 mM of AgNPs as well as free nanoparticles in tandem. The dilutions were prepared in injection water. A 30 µL sample was loaded in each well, and the zones of inhibition were measured after 24 h of incubation in an incubator (ECOCELL, Guwahat, India).

#### 3.12.3. Anticancer Activity

The samples of LC-AgNPs, plant extract, and free AgNPs were sent to the Central Lab of Inmol Cancer Hospital, Lahore, to scrutinize the anticancer potential. The samples were freshly prepared before being dispatched. The cell lines were obtained from Inmol Hospital and Cancer Research Centre, Lahore. The TPC1 cancerous cell lines were cultured in RPMI 1640 (with L-glutamate) containing 9.2% bovine serum albumin, 0.65% streptomycin and 1.1% amphotericine B.

The following formula was used for percentage cell viability:

$$\mathrm{Percentage}\;\mathrm{cell}\;\mathrm{viability}=\frac{Ae-An}{Ap-An}$$

where “*Ae*” is the absorbance of the experimental sample, and “*An*” and “*Ap*” are the absorbance of the negative and positive controls.

### 3.13. Statistical Analysis

All the investigations were carried out in triplicate with mean ± standard deviation. All plots were drawn using the Origin95 software. XRD was plotted using X’pert HighScore plus, and SEM sizing was completed using ImageJ software. The *p* value < 0.05 was considered significant [106].

## 4. Conclusions

AgNPs exhibiting biomedical potential (antibacterial, anticancer, and antioxidant) have earned much attention. Plants have proved themselves as a competitive source for AgNPs synthesis because they eliminate the requirement for hazardous chemicals (borohydride, 2-mercaptoethanol, citrate and thio-glycerol) used in chemical synthesis and offer a shorter synthesis time (as in the case of synthesis from microorganisms). *L. chinensis* is a medicinal plant and possesses effective antibacterial, anticancer potential in addition to being used in wound healing, cardiovascular, and kidney diseases. The extract of *L. chinensis* acts as a capping agent in AgNPs production. Moreover, alcohol, phenolic compounds, alkane, alkenes, amide, nitrites, ketones and alkyl groups were noticed to be accountable for silver reduction into AgNPs. LC-AgNPs exhibited antibacterial activity against all bacterial strains used. Furthermore, LC-AgNPs reported higher antioxidant activity than *L. chinensis* extract. Anticancer activity was estimated against the TPC1 cell line and LC-AgNPs were found to have the best cytotoxic activity. The crux of this research is that LC-AgNPs have effective antioxidant, antibacterial, and anticancer activity, proposing their biomedical application as a drug. The fruitful translation of these results into practical applications could significantly impact the biomedical field and improve healthcare outcomes by subsidizing the development of sustainable and eco-friendly nanomaterials. While AgNPs show promise as antibacterial, antioxidant, and anticancer agents, there are still some limitations to consider. Extensive research is needed to explore the exact underlying mechanisms that impart this potential to AgNPs. Moreover, while some studies show promising results, more research is needed to understand the underlying mechanisms and optimize the therapeutic potential.

## Figures and Tables

**Table 1:** FTIR spectrum peak analysis of *L. chinensis* extract and LC-AgNPs.

Sample	Wavelength (cm^−1^)	Bond Stretching	Functional Groups
Plant extract	616.06	C–Cl stretching	Alkyl group
	1634.98	C=O stretching	Ketones
	3332.17	OH stretching	Phenolic compound
LC-AgNPs	593.96	C–C–C–N bonding	Nitrites
	1062.30	C–H stretching	Cellulose
	1394.37	–C–H– Bending	Polyphenols
	1634.98	C=O stretching	Ketones
	2982.30	C–H stretching	Alkanes
	3336.47	C=C stretching	Alkenes

**Table 2:** DPPH radical scavenging activity of *L. chinensis* extract, LC-AgNPs, and ascorbic acid.

Samples	Concentrations (µg/mL)	Wavelength	
		515 nm	
		Scavenging Activity (%)	IC_50_ Value (µg/mL)
Plant extract	50	16.70 ± 1.13	209.44 ± 0.24
	100	25.08 ± 1.30	
	150	33.72 ± 1.22	
	200	45.94 ± 1.17	
	250	55.95 ± 1.51	
	300	68.00 ± 1.39	
LC-AgNPs	50	41.62 ± 1.30	85.01 ± 0.17
	100	50.25 ± 1.26	
	150	58.11 ± 1.04	
	200	69.94 ± 1.38	
	250	76.12 ± 1.22	
	300	84.24 ± 1.43	
Ascorbic acid (standard)	50	55.61 ± 1.35	47.63 ± 0.21
	100	64.37 ± 1.26	
	150	73.83 ± 1.12	
	200	81.26 ± 1.60	
	250	89.50 ± 1.38	
	300	91.84 ± 1.47	

**Table 3:** Diameter of zones of inhibition in mm (Mean ± SD) for different doses (µg/mL) of free and LC-AgNPs after 24 h.

Bacterial Strains	Various Conc. (mM)					
	3		4		5	
	AgNO_3_	LC-AgNPs	AgNO_3_	LC-AgNPs	AgNO_3_	LC-AgNPs
*E. coli*	9 ± 0.3	11 ± 0.5	8 ± 0.3	13 ± 0.4	10 ± 0.5	10 ± 0.4
*S. aureus*	12 ± 0.4	14 ± 0.4	14 ± 0.6	15 ± 0.3	14 ± 0.3	14 ± 0.3
*B. subtilis*	9 ± 0.4	8 ± 0.6	10 ± 0.4	10 ± 0.3	7 ± 0.3	9 ± 0.5
*P. aeruginosa*	9 ± 0.4	15 ± 0.7	9 ± 0.9	17 ± 0.7	8 ± 0.5	11 ± 0.5

All the cultures were obtained from the culture bank of the Institute of Industrial Biotechnology, GC University, Lahore. ± indicates the standard deviation.

**Table 4:** In vitro cell cycle analysis in TPC1 cells of human thyroid cancerous cells treated with various plant extract samples.

Exposure Time (min)	Various Samples vs. Dilutions (%)	Estimated No. of Cells/Cycle for Variable Cell Lines (%)		
		Plant Extract	Free AgNPs	LC-AgNPs
30	Control	88	88	88
30	10	52	63	46
	15	45	56	38
60	5	60	71	57
	10	64	76	61
90	5	76	82	70

## Data Availability

Data are contained within the article.

## References

[1] Ali B.A., Azeem M.A., Qayyum A., Mustafa G., Ahmad M.A., Javed M.T., Chaudhary H.J. (2021). Bio-Fabricated Silver Nanoparticles: A Sustainable Approach for Augmentation of Plant Growth and Pathogen Control. Sustainable Agriculture Reviews 53.

[2] Faryal S., Ullah R., Khan M.N., Ali B., Hafeez A., Jaremko M., Qureshi K.A. (2022). Thiourea-Capped Nanoapatites Amplify Osmotic Stress Tolerance in *Zea mays* L. by Conserving Photosynthetic Pigments, Osmolytes Biosynthesis and Antioxidant Biosystems. Molecules.

[3] Ahmed S., Ahmad M., Swami B.L., Ikram S. (2016). A review on plants extract mediated synthesis of silver nanoparticles for antimicrobial applications: A green expertise. J. Adv. Res..

[4] Saratale R.G., Benelli G., Kumar G., Kim D.S., Saratale G.D. (2018). Bio-fabrication of silver nanoparticles using the leaf extract of an ancient herbal medicine, dandelion (*Taraxacum officinale*), evaluation of their antioxidant, anticancer potential, and antimicrobial activity against phytopathogens. Environ. Sci. Pollut. Res..

[5] Mobin M., Ahmad I., Shoeb M. (2022). Investigation into the highly efficient Artemisia absinthium-silver nanoparticles composite as a novel environmentally benign corrosion inhibitor for mild steel in 1M HCl. J. Adhes. Sci. Technol..

[6] Basik M., Mobin M., Shoeb M. (2020). Cysteine-silver-gold Nanocomposite as potential stable green corrosion inhibitor for mild steel under acidic condition. Sci. Rep..

[7] Bourgonje C.R., da Silva D.R.C., McIlroy E., Calvert N.D., Shuhendler A.J., Scaiano J.C. (2023). Silver nanoparticles with exceptional near-infrared absorbance for photoenhanced antimicrobial applications. J. Mater. Chem. B.

[8] Elbagory A.M., Hull R., Meyer M., Dlamini Z. (2023). Reports of Plant-Derived Nanoparticles for Prostate Cancer Therapy. Plants.

[9] Shoeb M., Mashkoor F., Khan M.N., Anwer A.H., Ahmad S., Yi H., Jeong C. (2023). Unraveling the electrochemical properties and charge storage mechanisms of lactobacillus-mediated synthesized RGO-titanium silver nanocomposite as a promising binder-free electrode for asymmetric supercapacitor device. J. Alloys Compd..

[10] Khan R., Assad N., Naeem M., Sher M., Alatawi S., Alatawi M.S., Omran A.M.E., Jame R.M.A., Adnan M., Khan M.N. (2023). *Aconitum lycoctonum* L. (Ranunculaceae) mediated biogenic synthesis of silver nanoparticles as potential antioxidant, anti -inflammatory, antimicrobial and antidiabetic agents. BMC Chem..

[11] Kiani H.S., Ali B., Al-Sadoon M.K., Al-Otaibi H.S., Ali A. (2023). LC-MS/MS and GC-MS Identification of Metabolites from the Selected Herbs and Spices, Their Antioxidant, Anti-Diabetic Potential, and Chemometric Analysis. Processes.

[12] Ullah B., Hassan S., Khan M.N., Razzaq A., Al-Sadoon M.K., Wahab S., Kaplan A., Celikoglu U., Razak S.A., Ozdemir F.A. (2024). Phytochemical screening, antimicrobial, antipellicle and antibiofilm activities of the root of alpine medicinal herb (*Arnebia euchroma* (Royle) I.M.Johnst.). Pol. J. Environ. Stud..

[13] Kumkoon T., Srisaisap M., Boonserm P. (2023). Biosynthesized Silver Nanoparticles Using *Morus alba* (White Mulberry) Leaf Extract as Potential Antibacterial and Anticancer Agents. Molecules.

[14] Imran M., Iqbal A., Badshah S.L., Ahmad I., Shami A., Ali B., Alatawi F.S., Alatawi M.S., Mostafa Y.S., Alamri S.A. (2023). Exploring the hidden treasures of *Nitella hyalina*: A comprehensive study on its biological compounds, nutritional profile, and unveiling its antimicrobial, antioxidative, and hypoglycemic properties. World J. Microbiol. Biotechnol..

[15] Álvarez-Martínez F.J., Barrajón-Catalán E., Herranz-López M., Micol V. (2021). Antibacterial plant compounds, extracts and essential oils: An updated review on their effects and putative mechanisms of action. Phytomedicine.

[16] Avci H., Gergeroglu H. (2019). Synergistic effects of plant extracts and polymers on structural and antibacterial properties for wound healing. Polym. Bull..

[17] Rodríguez De Luna S.L., Ramírez-Garza R.E., Serna Saldívar S.O. (2020). Environmentally friendly methods for flavonoid extraction from plant material: Impact of their operating conditions on yield and antioxidant properties. Sci. World J..

[18] Merecz-Sadowska A., Sitarek P., Kucharska E., Kowalczyk T., Zajdel K., Cegliński T., Zajdel R. (2021). Antioxidant properties of plant-derived phenolic compounds and their effect on skin fibroblast cells. Antioxidants.

[19] Zhu H., Ahmad T., Zheng Y., Moosa A., Nie C., Liu Y. (2022). First report of leaf blight disease of *Livistona chinensis* caused by *Alternaria alternata* in Pakistan. Plant Dis..

[20] Iqbal M.J., Ali S., Rashid U., Kamran M., Malik M.F., Sughra K., Zeeshan N., Afroz A., Saleem J., Saghir M. (2018). Biosynthesis of silver nanoparticles from leaf extract of *Litchi chinensis* and its dynamic biological impact on microbial cells and human cancer cell lines. Cell Mol. Biol..

[21] Essien E.E., Antia B.S., Etuk E.I. (2017). Phytoconstituents, antioxidant and antimicrobial activities of *Livistona chinensis* (Jacquin), *Saribus rotundifolius* (Lam.) Blume and Areca catechu Linnaeus Nuts. Pharm. Biosci. J..

[22] Jabeen S., Ali M.F., Mohi ud Din A., Javed T., Mohammed N.S., Chaudhari S.K., Javed M.A., Ali B., Zhang L., Rahimi M. (2023). Phytochemical screening and allelopathic potential of phytoextracts of three invasive grass species. Sci. Rep..

[23] Hafeez A., Ali B., Javed M.A., Saleem A., Fatima M., Fathi A., Afridi M.S., Aydin V., Oral M.A., Soudy F.A. (2023). Plant breeding for harmony between sustainable agriculture, the environment, and global food security: An era of genomics-assisted breeding. Planta.

[24] Abeed A.H.A., AL-Huqail A.A., Albalawi S., Alghamdi S.A., Ali B., Alghanem S.M.S., Al-Haithloul H.A.S., Amro A., Tammam S.A., El-Mahdy M.T. (2023). Calcium nanoparticles mitigate severe salt stress in *Solanum lycopersicon* by instigating the antioxidant defense system and renovating the protein profile. S. Afr. J. Bot..

[25] Abeed A.H.A., Saleem M.H., Asghar M.A., Mumtaz S., Ameer A., Ali B., Alwahibi M.S., Elshikh M.S., Ercisli S., Elsharkawy M.M. (2023). Ameliorative Effects of Exogenous Potassium Nitrate on Antioxidant Defense System and Mineral Nutrient Uptake in Radish (*Raphanus sativus* L.) under Salinity Stress. ACS Omega.

[26] Alshegaihi R.M., Mfarrej M.F.B., Saleem M.H., Parveen A., Ahmad K.S., Ali B., Abeed A.H.A., Alshehri D., Alghamdi S.A., Alghanem S.M.S. (2023). Effective citric acid and EDTA treatments in cadmium stress tolerance in pepper (*Capsicum annuum* L.) seedlings by regulating specific gene expression. S. Afr. J. Bot..

[27] Tabassum S., Ahmad S., Ali B., Usman F., Jabeen Q., Sajid-ur-Rehman M., Ahmed M., Zubair H.M., Alkazmi L., Batiha G.E.-S. (2023). Chemical profiling and evaluation of toxicological, antioxidant, anti-inflammatory, anti-nociceptive and tyrosinase inhibitory potential of *Portulacaria afra* using in-vitro, in-vivo and in-silico studies. Arab. J. Chem..

[28] Ali Q., Shabaan M., Ashraf S., Kamran M., Zulfiqar U., Ahmad M., Zahir Z.A., Sarwar M.J., Iqbal R., Ali B. (2023). Comparative efficacy of different salt tolerant rhizobial inoculants in improving growth and productivity of *Vigna radiata* L. under salt stress. Sci. Rep..

[29] Ali B., Hafeez A., Afridi M.S., Javed M.A., Sumaira, Suleman F., Nadeem M., Ali S., Alwahibi M.S., Elshikh M.S. (2023). Bacterial-Mediated Salinity Stress Tolerance in Maize (*Zea mays* L.): A Fortunate Way toward Sustainable Agriculture. ACS Omega.

[30] Batool S., Batool S., Batool T., Iram F., Almas T., Faizan M., Assad N., Al-Sadoon M.K., Khan M.N., Ullah B. (2023). Delving the Role of the Ameliorative Effects of *Caralluma tuberculata* NE Br.(Apocynaceae) on Diabetes and Its Effect on the Organs Weight of Alloxan-Induced Adult Male Mice. Polish J. Environ. Stud..

[31] Khan S., Ullah H., Rahim F., Hussain R., Khan Y., Khan M.S., Iqbal R., Ali B., Albeshr M.F. (2023). Synthesis, biological evaluation and molecular docking study of pyrimidine based thiazolidinone derivatives as potential anti-urease and anti-cancer agents. J. Saudi Chem. Soc..

[32] Thakur A., Singh S., Singh N., Ali B., Hafeez A., Vodnar D.C., Marc R.A. (2022). Nutritional evaluation, phytochemical makeup, antibacterial and antioxidant properties of wild plants utilized as food by the Gaddis-a tribal tribe in the Western Himalayas. Front. Agron..

[33] Farrukh A., Khattak S.H., Kaleem I., Basheer S., Bangash S.A.K., Ali G.M., Khan M.N., Abul Farah M., Al-Anazi K.M., Kaplan A. (2023). Evaluation of Counteraction Potential of ZnO-NPs and/or *Piperacillin-Tazobactam* against Multi-Drug Resistant *Pseudomonas aeruginosa* and MCF-7 and HepG2 Cell Lines. Polish J. Environ. Stud..

[34] Moradi M.T., Karimi A., Shahrani M., Hashemi L., Ghaffari-Goosheh M.S. (2019). Anti-influenza virus activity and phenolic content of pomegranate (*Punica granatum* L.) peel extract and fractions. Avicenna J. Med. Biotechnol..

[35] Gali L., Bedjou F. (2019). Antioxidant and anticholinesterase effects of the ethanol extract, ethanol extract fractions and total alkaloids from the cultivated *Ruta chalepensis*. S. Afr. J. Bot..

[36] Suresh D., Nethravathi P.C., Udayabhanu, Rajanaika H., Nagabhushana H., Sharma S.C. (2015). Green synthesis of multifunctional zinc oxide (ZnO) nanoparticles using *Cassia fistula* plant extract and their photodegradative, antioxidant and antibacterial activities. Mater. Sci. Semicond. Process..

[37] Krishnan R.Y., Rajan K.S. (2016). Microwave assisted extraction of flavonoids from *Terminalia bellerica*: Study of kinetics and thermodynamics. Sep. Purif. Technol..

[38] Liang Q., Chen H., Zhou X., Deng Q., Hu E., Zhao C., Gong X. (2017). Optimized microwave-assistant extraction combined ultrasonic pretreatment of flavonoids from *Periploca forrestii* Schltr. and evaluation of its anti-allergic activity. Electrophoresis.

[39] Yerena-Prieto B.J., Gonzalez-Gonzalez M., Vázquez-Espinosa M., González-de-Peredo A.V., García-Alvarado M.Á., Palma M., Rodríguez-Jimenes G.d.C., Barbero G.F. (2022). Optimization of an ultrasound-assisted extraction method applied to the extraction of flavonoids from Moringa leaves (*Moringa oleífera* Lam.). Agronomy.

[40] Pimentel-Moral S., Borrás-Linares I., Lozano-Sánchez J., Arráez-Román D., Martínez-Férez A., Segura-Carretero A. (2018). Microwave-assisted extraction for Hibiscus sabdariffa bioactive compounds. J. Pharm. Biomed. Anal..

[41] Okiyama D.C.G., Soares I.D., Cuevas M.S., Crevelin E.J., Moraes L.A.B., Melo M.P., Oliveira A.L., Rodrigues C.E.C. (2018). Pressurized liquid extraction of flavanols and alkaloids from cocoa bean shell using ethanol as solvent. Food Res. Int..

[42] Erşan S., Üstündağ Ö.G., Carle R., Schweiggert R.M. (2018). Subcritical water extraction of phenolic and antioxidant constituents from pistachio (*Pistacia vera* L.) hulls. Food Chem..

[43] Link S., Yrjas P., Lindberg D., Trikkel A. (2022). Characterization of ash melting of reed and wheat straw blend. ACS omega.

[44] Das C.M., Yang F., Yang Z., Liu X., Hoang Q.T., Xu Z., Neermunda S., Kong K.V., Ho H., Ju L.A. (2023). Computational Modeling for Intelligent Surface Plasmon Resonance Sensor Design and Experimental Schemes for Real-Time Plasmonic Biosensing: A Review. Adv. Theory Simul..

[45] Nagime P.V., Singh S., Shaikh N.M., Gomare K.S., Chitme H., Abdel-Wahab B.A., Alqahtany Y.S., Khateeb M.M., Habeeb M.S., Bakir M.B. (2023). Biogenic Fabrication of Silver Nanoparticles Using *Calotropis procera* Flower Extract with Enhanced Biomimetics Attributes. Materials.

[46] Nduati T.W., Wagara I.N., Walyambillah W., Were B., Matasyoh J.C. (2023). Bioactive compounds from *Juniperus procera* (Cupressaceae) with activity against common bean bacterial pathogens. Afr. J. Biotechnol..

[47] Taha M., Elazab S.T., Abdelbagi O., Saati A.A., Babateen O., Baokbah T.A.S., Qusty N.F., Mahmoud M.E., Ibrahim M.M., Badawy A.M. (2023). Phytochemical analysis of *Origanum majorana* L. extract and investigation of its antioxidant, anti-inflammatory and immunomodulatory effects against experimentally induced colitis downregulating Th17 cells. J. Ethnopharmacol..

[48] El-Naggar N.E.-A., Hussein M.H., El-Sawah A.A. (2017). Bio-fabrication of silver nanoparticles by phycocyanin, characterization, in vitro anticancer activity against breast cancer cell line and in vivo cytotxicity. Sci. Rep..

[49] Yu C., Tang J., Liu X., Ren X., Zhen M., Wang L. (2019). Green Biosynthesis of Silver Nanoparticles Using *Eriobotrya japonica* (Thunb.) Leaf Extract for Reductive Catalysis. Materials.

[50] Salayová A., Bedlovičová Z., Daneu N., Baláž M., Lukáčová Bujňáková Z., Balážová Ľ., Tkáčiková Ľ. (2021). Green synthesis of silver nanoparticles with antibacterial activity using various medicinal plant extracts: Morphology and antibacterial efficacy. Nanomaterials.

[51] Manikandan E., Krishnan V. (2016). Green Synthesis of Silver Nanoparticles Using Piper nigrum Concoction and its Anticancer Activity against MCF-7 and Hep-2 Cell Lines. J. Antimicrob. Agents.

[52] He Y., Wei F., Ma Z., Zhang H., Yang Q., Yao B., Huang Z., Li J., Zeng C., Zhang Q. (2017). Green synthesis of silver nanoparticles using seed extract of *Alpinia katsumadai*, and their antioxidant, cytotoxicity, and antibacterial activities. RSC Adv..

[53] Jain S., Mehata M.S. (2017). Medicinal Plant Leaf Extract and Pure Flavonoid Mediated Green Synthesis of Silver Nanoparticles and their Enhanced Antibacterial Property. Sci. Rep..

[54] Oves M., Ahmar Rauf M., Aslam M., Qari H.A., Sonbol H., Ahmad I., Sarwar Zaman G., Saeed M. (2022). Green synthesis of silver nanoparticles by *Conocarpus Lancifolius* plant extract and their antimicrobial and anticancer activities. Saudi J. Biol. Sci..

[55] Sadeghi B., Gholamhoseinpoor F. (2015). A study on the stability and green synthesis of silver nanoparticles using *Ziziphora tenuior* (Zt) extract at room temperature. Spectrochim. Acta—Part A Mol. Biomol. Spectrosc..

[56] David S.A., Ponvel K.M., Fathima M.A., Anita S., Ashli J., Athilakshmi A. (2014). Biosynthesis of silver nanoparticles by *Momordica charantia* leaf extract: Characterization and their antimicrobial activities. J. Nat. Prod. Plant Resour..

[57] Liu Y.-S., Chang Y.-C., Chen H.-H. (2018). Silver nanoparticle biosynthesis by using phenolic acids in rice husk extract as reducing agents and dispersants. J. Food Drug Anal..

[58] Manikandan D.B., Sridhar A., Krishnasamy Sekar R., Perumalsamy B., Veeran S., Arumugam M., Karuppaiah P., Ramasamy T. (2021). Green fabrication, characterization of silver nanoparticles using aqueous leaf extract of *Ocimum americanum* (Hoary Basil) and investigation of its in vitro antibacterial, antioxidant, anticancer and photocatalytic reduction. J. Environ. Chem. Eng..

[59] Pungle R., Nile S.H., Makwana N., Singh R., Singh R.P., Kharat A.S. (2022). Green Synthesis of Silver Nanoparticles Using the *Tridax procumbens* Plant Extract and Screening of Its Antimicrobial and Anticancer Activities. Oxidative Med. Cell. Longev..

[60] Shyamalagowri S., Charles P., Manjunathan J., Kamaraj M., Anitha R., Pugazhendhi A. (2022). In vitro anticancer activity of silver nanoparticles phyto-fabricated by *Hylocereus undatus* peel extracts on human liver carcinoma (HepG2) cell lines. Process Biochem..

[61] Bharadwaj K.K., Rabha B., Pati S., Choudhury B.K., Sarkar T., Gogoi S.K., Kakati N., Baishya D., Kari Z.A., Edinur H.A. (2021). Green synthesis of silver nanoparticles using *Diospyros malabarica* fruit extract and assessments of their antimicrobial, anticancer and catalytic reduction of 4-nitrophenol (4-NP). Nanomaterials.

[62] Giri A.K., Jena B., Biswal B., Pradhan A.K., Arakha M., Acharya S., Acharya L. (2022). Green synthesis and characterization of silver nanoparticles using *Eugenia roxburghii* DC. extract and activity against biofilm-producing bacteria. Sci. Rep..

[63] Ashraf H., Anjum T., Riaz S., Naseem S. (2020). Microwave-assisted green synthesis and characterization of silver nanoparticles using *Melia azedarach* for the management of *Fusarium* wilt in tomato. Front. Microbiol..

[64] Chandraker S.K., Lal M., Khanam F., Dhruve P., Singh R.P., Shukla R. (2022). Therapeutic potential of biogenic and optimized silver nanoparticles using *Rubia cordifolia* L. leaf extract. Sci. Rep..

[65] Tormena R.P.I., Rosa E.V., Mota B.d.F.O., Chaker J.A., Fagg C.W., Freire D.O., Martins P.M., da Silva I.C.R., Sousa M.H. (2020). Evaluation of the antimicrobial activity of silver nanoparticles obtained by microwave-assisted green synthesis using *Handroanthus impetiginosus* (Mart. ex DC.) Mattos underbark extract. RSC Adv..

[66] Elamawi R.M., Al-Harbi R.E., Hendi A.A. (2018). Biosynthesis and characterization of silver nanoparticles using *Trichoderma longibrachiatum* and their effect on phytopathogenic fungi. Egypt. J. Biol. Pest Control.

[67] Thirumagal N., Jeyakumari A.P. (2020). Structural, optical and antibacterial properties of green synthesized silver nanoparticles (AgNPs) using *Justicia adhatoda* L. leaf extract. J. Clust. Sci..

[68] Jyoti K., Singh A., Fekete G., Singh T. (2020). Cytotoxic and radiosensitizing potential of silver nanoparticles against HepG-2 cells prepared by biosynthetic route using *Picrasma quassioides* leaf extract. J. Drug Deliv. Sci. Technol..

[69] Ghabban H., Alnomasy S.F., Almohammed H., Al Idriss O.M., Rabea S., Eltahir Y. (2022). Antibacterial, Cytotoxic, and Cellular Mechanisms of Green Synthesized Silver Nanoparticles against Some Cariogenic Bacteria (*Streptococcus mutans* and *Actinomyces viscosus*). J. Nanomater..

[70] Sana S.S., Dogiparthi L.K. (2018). Green synthesis of silver nanoparticles using *Givotia moluccana* leaf extract and evaluation of their antimicrobial activity. Mater. Lett..

[71] Rajendran R., Ganesan N., Balu S.K., Alagar S., Thandavamoorthy P., Thiruvengadam D. (2015). Green synthesis, characterization, antimicrobial and cytotoxic effects of silver nanoparticles using *Origanum heracleoticum* L. leaf extract. Int. J. Pharm. Pharm. Sci..

[72] Kumar V., Singh S., Srivastava B., Bhadouria R., Singh R. (2019). Green synthesis of silver nanoparticles using leaf extract of *Holoptelea integrifolia* and preliminary investigation of its antioxidant, anti-inflammatory, antidiabetic and antibacterial activities. J. Environ. Chem. Eng..

[73] Rajakumar G., Gomathi T., Thiruvengadam M., Rajeswari V.D., Kalpana V.N., Chung I.-M. (2017). Evaluation of anti-cholinesterase, antibacterial and cytotoxic activities of green synthesized silver nanoparticles using from *Millettia pinnata* flower extract. Microb. Pathog..

[74] Ravichandran V., Vasanthi S., Shalini S., Ali Shah S.A., Harish R. (2016). Green synthesis of silver nanoparticles using *Atrocarpus altilis* leaf extract and the study of their antimicrobial and antioxidant activity. Mater. Lett..

[75] Kumar B., Smita K., Seqqat R., Benalcazar K., Grijalva M., Cumbal L. (2016). In vitro evaluation of silver nanoparticles cytotoxicity on Hepatic cancer (Hep-G2) cell line and their antioxidant activity: Green approach for fabrication and application. J. Photochem. Photobiol. B Biol..

[76] Baharara J., Ramezani T., Mousavi M., Asadi-Samani M. (2017). Antioxidant and anti-inflammatory activity of green synthesized silver nanoparticles using Salvia officinalis extract. Ann. Trop. Med. Public Health.

[77] Tyagi P.K., Tyagi S., Gola D., Arya A., Ayatollahi S.A., Alshehri M.M., Sharifi-Rad J. (2021). Ascorbic acid and polyphenols mediated green synthesis of silver nanoparticles from *Tagetes erecta* L. aqueous leaf extract and studied their antioxidant properties. J. Nanomater..

[78] Fierascu R.C., Bunghez I.R., Somoghi R., Fierascu I., Ion R.M. (2014). Characterization of silver nanoparticles obtained by using *Rosmarinus officinalis* extract and their antioxidant activity. Rev. Roum. Chim..

[79] Rajakannu S., Shankar S., Perumal S., Subramanian S., Dhakshinamoorthy G.P. (2015). Biosynthesis of silver nanoparticles using Garcinia mangostana fruit extract and their antibacterial, antioxidant activity. Int. J. Curr. Microbiol. Appl. Sci.

[80] Al-Shmgani H.S.A., Mohammed W.H., Sulaiman G.M., Saadoon A.H. (2017). Biosynthesis of silver nanoparticles from *Catharanthus roseus* leaf extract and assessing their antioxidant, antimicrobial, and wound-healing activities. Artif. Cells Nanomed. Biotechnol.

[81] Qais F.A., Shafiq A., Khan H.M., Husain F.M., Khan R.A., Alenazi B., Alsalme A., Ahmad I. (2019). Antibacterial effect of silver nanoparticles synthesized using *Murraya koenigii* (L.) against multidrug-resistant pathogens. Bioinorg. Chem. Appl..

[82] Younas M., Rasool M.H., Khurshid M., Khan A., Nawaz M.Z., Ahmad I., Lakhan M.N. (2023). *Moringa oleifera* leaf extract mediated green synthesis of silver nanoparticles and their antibacterial effect against selected gram-negative strains. Biochem. Syst. Ecol..

[83] Breijyeh Z., Jubeh B., Karaman R. (2020). Resistance of gram-negative bacteria to current antibacterial agents and approaches to resolve it. Molecules.

[84] Qamer S., Romli M.H., Che-Hamzah F., Misni N., Joseph N.M.S., Al-Haj N.A., Amin-Nordin S. (2021). Systematic review on biosynthesis of silver nanoparticles and antibacterial activities: Application and theoretical perspectives. Molecules.

[85] Some S., Sen I.K., Mandal A., Aslan T., Ustun Y., Yilmaz E.Ş., Katı A., Demirbas A., Mandal A.K., Ocsoy I. (2018). Biosynthesis of silver nanoparticles and their versatile antimicrobial properties. Mater. Res. Express.

[86] Rajeshkumar S., Bharath L. (2017). V Mechanism of plant-mediated synthesis of silver nanoparticles—A review on biomolecules involved, characterisation and antibacterial activity. Chem. Biol. Interact..

[87] Farshori N.N., Al-Oqail M.M., Al-Sheddi E.S., Al-Massarani S.M., Saquib Q., Siddiqui M.A., Wahab R., Al-Khedhairy A.A. (2022). Green synthesis of silver nanoparticles using *Phoenix dactylifera* seed extract and its anticancer effect against human lung adenocarcinoma cells. J. Drug Deliv. Sci. Technol..

[88] Saber M.M., Mirtajani S.B., Karimzadeh K. (2018). Green synthesis of silver nanoparticles using *Trapa natans* extract and their anticancer activity against A431 human skin cancer cells. J. Drug Deliv. Sci. Technol..

[89] Anandan M., Poorani G., Boomi P., Varunkumar K., Anand K., Chuturgoon A.A., Saravanan M., Gurumallesh Prabu H. (2019). Green synthesis of anisotropic silver nanoparticles from the aqueous leaf extract of *Dodonaea viscosa* with their antibacterial and anticancer activities. Process Biochem..

[90] Alahmad A., Feldhoff A., Bigall N.C., Rusch P., Scheper T., Walter J.-G. (2021). *Hypericum perforatum* L.-Mediated Green Synthesis of Silver Nanoparticles Exhibiting Antioxidant and Anticancer Activities. Nanomaterials.

[91] Alharbi N.S., Alsubhi N.S. (2022). Green synthesis and anticancer activity of silver nanoparticles prepared using fruit extract of *Azadirachta indica*. J. Radiat. Res. Appl. Sci..

[92] Gomathi A.C., Rajarathinam S.R.X., Sadiq A.M., Rajeshkumar S. (2020). Anticancer activity of silver nanoparticles synthesized using aqueous fruit shell extract of Tamarindus indica on MCF-7 human breast cancer cell line. J. Drug Deliv. Sci. Technol..

[93] Narasimha V.R., Latha T.S., Pallu R., Panati K., Narala V.R. (2022). Anticancer activities of biogenic silver nanoparticles targeting apoptosis and inflammatory pathways in colon cancer cells. J. Clust. Sci..

[94] El Raey M.A., El-Hagrassi A.M., Osman A.F., Darwish K.M., Emam M. (2019). *Acalypha wilkesiana* flowers: Phenolic profiling, cytotoxic activity of their biosynthesized silver nanoparticles and molecular docking study for its constituents as Topoisomerase-I inhibitors. Biocatal. Agric. Biotechnol..

[95] Rajawat S., Malik M.M. (2019). Anticancer activity of green silver nanoparticles against He-La cervical cancer cell lines. Mater. Today Proc..

[96] Yadav A., Mendhulkar V.D. (2018). Antiproliferative activity of Camellia sinensis mediated silver nanoparticles on three different human cancer cell lines. J. Cancer Res. Ther..

[97] Datkhile K.D., Durgawale P.P., Patil M.N. (2017). Biogenic silver nanoparticles are equally Cytotoxic as Chemically Synthesized silver nanoparticles. Biomed. Pharmacol. J..

[98] Lydia E., John S., Mohammed R., Sivapriya T. (2016). Investigation on the Phytochemicals present in the Fruit peel of *Carica papaya* and evaluation of its Antioxidant and Antimicrobial property. Res. J. Pharmacogn. Phytochem..

[99] De Caro C., Claudia H. (2015). UV/VIS Spectrophotometry—Fundamentals and Applications.

[100] Khanal L.N., Sharma K.R., Paudyal H., Parajuli K., Dahal B., Ganga G.C., Pokharel Y.R., Kalauni S.K. (2022). Green Synthesis of Silver Nanoparticles from Root Extracts of *Rubus ellipticus* Sm. and Comparison of Antioxidant and Antibacterial Activity. J. Nanomater..

[101] Velusamy P., Das J., Pachaiappan R., Vaseeharan B., Pandian K. (2015). Greener approach for synthesis of antibacterial silver nanoparticles using aqueous solution of neem gum (*Azadirachta indica* L.). Ind. Crops Prod..

[102] Clogston J.D., Patri A.K., McNeil S.E. (2011). Zeta Potential Measurement. Characterization of Nanoparticles Intended for Drug Delivery.

[103] Liu Y., Hou C., Jiao T., Song J., Zhang X., Xing R. (2018). Self-Assembled AgNP-Containing Nanocomposites Constructed by Electrospinning as Efficient Dye Photocatalyst Materials for Wastewater Treatment. Nanomaterials.

[104] Kharat S.N., Mendhulkar V.D. (2016). Synthesis, characterization and studies on antioxidant activity of silver nanoparticles using *Elephantopus scaber* leaf extract. Mater. Sci. Eng. C.

[105] Pungle R., Nile S.H., Kharat A.S. (2023). Green synthesis and characterization of *Solanum xanthocarpum* capped silver nanoparticles and its antimicrobial effect on multidrug-resistant bacterial (MDR) isolates. Chem. Biol. Drug Des..

[106] Dixon W.J., Massey F.J. (1951). Introduction to Statistical Analysis.

